# Reduced Sodium Portions Favor Osteogenic Properties and Cytocompatibility of 45S5-Based Bioactive Glass Particles

**DOI:** 10.3390/biomimetics8060472

**Published:** 2023-10-02

**Authors:** Stefanos Tsitlakidis, Frederike Hohenbild, Merve Saur, Arash Moghaddam, Elke Kunisch, Tobias Renkawitz, Isabel Gonzalo de Juan, Fabian Westhauser

**Affiliations:** 1 Department of Orthopaedics, Heidelberg University Hospital, Schlierbacher Landstraße 200a, 69118 Heidelberg, Germany; stefanos.tsitlakidis@med.uni-heidelberg.de (S.T.); merve.saur@med.uni-heidelberg.de (M.S.); elke.kunisch@med.uni-heidelberg.de (E.K.);; 2 PrivatÄrztliches Zentrum Aschaffenburg, Frohsinnstraße 12, 63739 Aschaffenburg, Germany; info@profmoghaddam.com; 3 Institut für Materialwissenschaft, Technische Universität Darmstadt, Otto-Berndt-Straße 3, 64287 Darmstadt, Germany

**Keywords:** sodium-reduced bioactive glass, 45S5 bioactive glass, sol gel, bone tissue engineering, human mesenchymal stromal cells, osteogenic differentiation, cell viability

## Abstract

Besides its favorable biological properties, the release of sodium (Na) from the well-known 45S5-bioactive glass (BG) composition (in mol%: 46.1, SiO_2_, 24.5 CaO, 24.5 Na_2_O, 6.0 P_2_O_5_) can hamper its cytocompatibility. In this study, particles of Na-reduced variants of 45S5-BG were produced in exchange for CaO and P_2_O_5_ via the sol-gel-route resulting in Na contents of 75%, 50%, 25% or 0% of the original composition. The release of ions from the BGs as well as their impact on the cell environment (pH values), viability and osteogenic differentiation (activity of alkaline phosphatase (ALP)), the expression of osteopontin and osteocalcin in human bone-marrow-derived mesenchymal stromal cells in correlation to the Na-content and ion release of the BGs was assessed. The release of Na-ions increased with increasing Na-content in the BGs. With decreasing Na content, the viability of cells incubated with the BGs increased. The Na-reduced BGs showed elevated ALP activity and a pro-osteogenic stimulation with accelerated osteopontin induction and a pronounced upregulation of osteocalcin. In conclusion, the reduction in Na-content enhances the cytocompatibility and improves the osteogenic properties of 45S5-BG, making the Na-reduced variants of 45S5-BG promising candidates for further experimental consideration.

## 1. Introduction

Synthetic bone substitutes and bioactive glasses (BG) in particular have gained importance concerning the surgical treatment of bone defects [1]. Still, autologous bone grafting, e.g., harvested from the iliac crests, represents the gold standard in bone defect augmentation [2]. However, using autologous bone grafts for bone defect treatment is associated with different challenges, such as donor site morbidity and quantitative limitations [3]. The use of synthetic bone substitutes might reduce or even replace the application of autologous bone in defect treatment. In this context, 45S5-BG (composition in mol%: 46.1 SiO_2_, 24.5 CaO, 24.5 Na_2_O, 6.0 P_2_O_5_) represents the most investigated BG and functions as a reference in order to compare newly developed BG compositions [4,5,6,7]. A variety of advantageous properties, such as adherence to the surrounding tissue in-vivo and the induction of osteogenic differentiation in bone precursor cells imparted by liberation of bioactive ions were identified for 45S5-BG [1,8,9,10]. Besides these favorable properties, mediated by its high initial bioreactivity, 45S5-BG generates a challenging biological environment by a burst release of sodium-ions (Na) that causes a pH-shift and an alkalization of the local environment [11,12,13,14,15,16]. These chemical processes might limit the cytocompatibility of the 45S5-BG composition. Furthermore, the effects of Na reduction or even of Na-free BGs have been evaluated in the past, particularly demonstrating superior properties with respect to cell viability, proliferation and osteogenic differentiation [11]. However, to our knowledge, specific characteristics of custom-made Na-reduced variants specifically based on 45S5-BG, such as ion release patterns, extracellular pH and their effects on cytocompatibility and osteogenic differentiation, have not been investigated so far.

In this study, Na contents of 45S5-BG were reduced to 75%, 50%, 25% and 0% of the original content in the 45S5-BG composition via the sol–gel approach in order to reduce the alkalization of the BG-cell interface to improve cytocompatibility. Typically, BGs can be prepared via a traditional melt-quenching route and sol–gel synthesis [17]. Compared to the traditional approach, the sol-gel route allows for obtaining BG powders with a higher specific surface area, which facilitates a higher level of bioactivity, through a low temperature synthesis technique. Moreover, the sol–gel approach enables the production of BG powders with a wider compositional range. For example, sol–gel-derived silicate glasses may contain up to 90 mol% of SiO_2_, while in melt-derived silicate glasses the SiO_2_ amount is limited to 60 mol% [18,19,20].

In this study, the mentioned modified BG-compositions and unmodified 45S5-BG were introduced to a cell culture setting of human-bone-marrow-derived mesenchymal stromal cells (BMSCs) in order to assess the impact of Na reduction in BG composition on cytocompatibility and osteogenic differentiation. Furthermore, the ion release profiles of the BG compositions and the BGs’ impact on extracellular pH were evaluated in order to assess the actual biological impact of ion release from the different BG compositions.

## 2. Materials and Methods

### 2.1. Study Ethics and Cell Origin

BMSCs of a 20-year-old male patient who underwent surgical treatment at the proximal femur at the Heidelberg Orthopedic University Hospital were harvested. Written informed consent was obtained prior to the collection of the cells and the study was approved by the responsible ethics committee of the Medical Faculty of Heidelberg University (S-340/2018).

### 2.2. Materials and Synthesis of Sol-Gel Bioactive Particles

Bioactive silicate powders based on the 45S5 system (SiO_2_-Na_2_O-CaO-P_2_O_5_) with different sodium contents were prepared by a sol–gel approach published previously [21]. In brief, tetraethyl orthosilicate (TEOS) was added to nitric acid 2M and distilled water at molar ratio H_2_O:TEOS of 12. Then, 100 mL of ethyl alcohol (≥99.8%, Carl Roth, Germany) were added to the mixture, and it was stirred for 1 h with magnetic stirring at room temperature. After that, the rest of the reagents were added to the hydrolyzed TEOS one by one in the following order: diammonium hydrogen phosphate ((NH_4_)_2_HPO_4_)), calcium nitrate tetrahydrate (Ca(NO_3_)_2_∙4H_2_O) and sodium nitrate (NaNO_3_). Upon adding each reagent, the mixture was stirred for 1 h before the next one was added. An ammonia solution (2 M) was added to the mixture drop-by-drop under continuous magnetic stirring to maintain a pH value of 10.5. The precipitate was stirred for 48 h at room temperature and aged at 60 °C over 24 h. The obtained white solid was separated from the mother waters by centrifugation at 4000 rpm and washed with distilled water three times. Finally, the BG nanoparticles were calcinated at 700 °C for 3 h in air. Unless otherwise stated, these above-mentioned reagents were all ≥98% pure, obtained from Sigma–Aldrich (Sigma-Aldrich, Steinheim, Germany), and were used as received. The glass compositions listed in Table 1 were selected on the basis of the following criteria: (i) the systematic substitution of Na_2_O by CaO and (ii) maintaing a CaO:P_2_O_5_ ratio lower than 4 to assure a low silicate network connectivity.

### 2.3. Physicochemical Characterization: XRD, Particle Size, and Zeta Potential

The crystalline structures of the as-synthesized materials were determined by x-ray powder diffraction (XRD). XRD patterns were recorded from 5° to 30° 2θ range, in a STADI P X diffractometer (STOE&Cie) with Mo Kα radiation at 45 kV voltage and 30 mA current. The phase analysis of the samples was achieved with the Match! software (Version 3.15; Crystal Impact, Bonn, Germany) by comparing the powder diffraction data with reference patterns. Z-potential measurements were performed in a Zetasizer Nano ZS (Malvern Instruments, Malvern, UK) at 25 °C. Since the diameter of the particles was less than 1 micrometer, Smoluchowski’s equation was applied to calculate the zeta potential from the measured electrophoretic mobilities [22]. In order to determine the surface charge of the as-synthesized materials, the mobility measurements were carried out in less than 5 min to avoid chemical transformations on their surface. The size characterization of the samples was performed by dynamic light scattering (DLS) also using the Zetasizer Nano ZS instrument. DLS means were carried out with a backscattering detection angle of 173° and a wavelength of 632.8 nm provided by a He-Ne laser. The samples for zeta potential and size measurements were prepared as follows: 5 mg of sample was suspended in 10 mL of distilled water followed by three times 1 min ultrasonication and 30 s rest before measuring.

### 2.4. Ion Release from as-Synthetized BG Particles

To measure the cumulative concentrations of ions liberated from the BGs, they were incubated at 37 °C in UltraPure DNase/RNase-Free Distilled Water (Life Technologies, Darmstadt, Germany) at a concentration of 10 mg/mL for 1, 7, 14 and 21 days. After 1, 7, 14 and 21 days the supernatants were filtered using a 0.45 µm sterile filter (Merck, Darmstadt, Germany) and acidified by the addition of 50 µL concentrated nitric acid to each sample of 5 mL. The supernatants were filtered using a 0.45 µm sterile filter (Merck, Darmstadt, Germany) and acidified by the addition of 50 µL concentrated nitric acid to each sample of 5 mL. Using an inductively coupled plasma-optical emission spectrophotometer (Agilent 720 ICP-OES; Agilent, Santa Clara, CA, USA) the release of Na, silicon (Si), calcium (Ca) and phosphorous (P) ions was measured. Samples were evaluated in triplicate.

### 2.5. BMSC Isolation, Cultivation and Characterization

BMSCs were obtained from bone marrow washouts and isolated as described previously [7,23,24]. In short, after collection in a heparinized syringe, the bone marrow was fractioned on a Ficoll-Paque Plus density gradient (GE Healthcare Europe, Freiburg, Germany). The fraction of mononuclear cells that contains the BMSCs was washed in PBS (Life Technologies) and seeded in T75 cell culture flasks (Nunc, Roskilde, Denmark) coated with 0.1% gelatin (Sigma–Aldrich, Steinheim, Germany). An expansion medium composed of Dulbecco’s Modified Eagle’s Medium (DMEM) high glucose supplemented with 12.5% Fetal Calf Serum (FCS), 1% L-glutamine, 1% non-essential amino acids (NEAA; all Life Technologies), 1% penicillin/streptomycin (Biochrom, Berlin, Germany), 0.1% β-mercaptoethanol (Life Technologies) and 4 ng/mL fibroblast growth factor 2 (Abcam, Cambridge, UK) was used for cell cultivation. Media were changed after the first 24 h to discard non-adherent cells and subsequently twice weekly. Cells were passaged at 80% confluency until usage in passages 4 and 5.

### 2.6. General Experimental Design: Overview

BMSCs were introduced to direct co-culture with the variants of 45S5-BG (Table 1). The BGs were added to cell culture the medium (CCM: 89% DMEM, 10% FCS, 1% penicillin/streptomycin) at a concentration of 1 mg/mL and placed in 24- and 96-well culture plates (both Nunc, Roskilde, Denmark). Cells were added achieving a cell density of 2 × 10^4^ cells per cm^2^. A control group was cultured in BG-free CCM. Media were changed twice weekly. Each group was composed of five biological replicates. After 1 (D1), 7 (D7), 14 (D14) and 21 (D21) days of direct co-culture, the pH values of the media were assessed, and cell viability was measured using a fluorescence-based cell viability assay. Osteogenic differentiation, representatively measured by alkaline phosphatase (ALP) activity and the expression of genes encoding for proteins of the osseous extracellular matrix (ECM), was evaluated on D1, D7, D14 and D21.

### 2.7. Measurement of pH Values

To analyze the influence of the different BG compositions on the pH value in the surrounding medium, cells were cultivated in 1 mL CCM per well containing different BGs while a control group was cultivated in BG-free CCM. Cultivation was performed in 24-well culture plates (Sarstedt, Nümbrecht, Germany). As for a reliable pH measurement, a minimal volume of 3 mL was necessary; all samples were prepared in threefold number. On the day before the pH evaluation 15 mL tubes (Merck) were stored in an incubator at 37 °C and 5% CO_2_ to level CO_2_ contents. On D1, D7, D14 and D21 the supernatants of all groups were collected and transferred to the prepared tubes. In this step, the supernatants of three wells were merged into one falcon to obtain the necessary volume for the pH measurement. Tubes were left open in the incubator for 5 min, and subsequently pH was evaluated using a benchtop pH meter (Sartorius, Göttingen, Germany). Additionally, the pH of the cell- and BG-free CCM was measured to function as a baseline.

### 2.8. Combined Evaluation of Cell Viability and Alkaline Phosphatase (ALP) Activity as a Marker of Osteogenic Differentiation

Viable cells were quantified by a Fluorescein Diacetate (FDA; Sigma-Aldrich) staining. The non-fluorescent FDA freely passes the cell membrane and is intracellularly hydrolyzed to fluorescein, whose fluorescence intensity correlates with the cell viability [9,25]. From the same cell lysates in a 96-well culture plate, ALP activity was analyzed using the fluorogenic substrate 4’Methylumbelliferone-phosphate (4-MUP; Thermo Fisher, Dreieich, Germany) following a recently published protocol [5]. In short, cells were washed in PBS, and the supernatant was discarded. The FDA stock solution (FDA in acetone at 100 µg/mL) was diluted in PBS to a final FDA concentration of 2 µg/mL. 100 µL of the working solution was added to each well and incubated at 37 °C for 5 min. The supernatant was discarded, and cells were washed in PBS. Lysis was performed using 150 µL 0.5% Triton-X-100 (Sigma–Aldrich) per well and incubating the cells at 37 °C for 5 min. 50 µL of the lysates were transferred to the empty wells of a white 96-well culture plate (Kisker Biotech, Steinfurt, Germany) and 100 µL of 100 µM 4-MUP in 1.5× ALP assay buffer (50 mM Tris pH 9.3 (Carl Roth, Karlsruhe, Germany), 1 mM MgCl2, 0.5 mM ZnCl2 (both Merck)) was added. Incubation occurred for 20 min at 37 °C before fluorescence intensity was quantified in a Wallac 1420 Victor microplate reader (Perkin Elmer, Waltham, MA, USA). The green fluorescent fluorescein was measured at 485/530 nm, followed by the measurement of the blue fluorescent 4-MU at 360/440 nm. Absolute ALP activity was determined via a standard of shrimp alkaline phosphatase (Thermo Fisher), and values of each sample were normalized to the determined cell viability.

### 2.9. qPCR of Genes Encoding for ECM Proteins

Using the PureLink RNA Mini Kit (Life Technologies), total RNA was isolated according to the manufacturer’s instructions. The amount and quality of extracted RNA were analyzed photometrically, employing a Nanodrop spectrophotometer (Thermo Fisher), and cDNA synthesis was conducted with the High-Capacity RNA-to-cDNA Kit (Life Technologies) following the manufacturer’s protocol. The samples were subjected to real-time quantitative (RT-q) PCR using SYBR Green Master Mix (Life Technologies). Two target genes, namely osteocalcin (*OCN*) and osteopontin (*OPN*), and one endogenous reference gene (14-3-3 protein zeta/delta; *YWHAZ*) were analyzed. The genes and respective primers are listed in Table 2. Using the ΔΔCt method, relative expression was calculated by relating each target gene to the reference gene and normalizing the expression to the control group.

### 2.10. Statistics

Statistical analyses were performed using IBM SPSS Statistics (Version 25; IBM, Armonk, NY, USA). Values were tested via one-way ANOVA followed by Bonferroni’s post-hoc test accepting *p*-values of <0.05 as significant. Graphs were designed with GraphPad Prism (Version 8.1.0; GraphPad Software, La Jolla, CA, USA). Values are shown as rounded means with standard deviation where applicable.

## 3. Results

### 3.1. Physicochemical Characterization of the as-Prepared Materials

**Figure 1:**
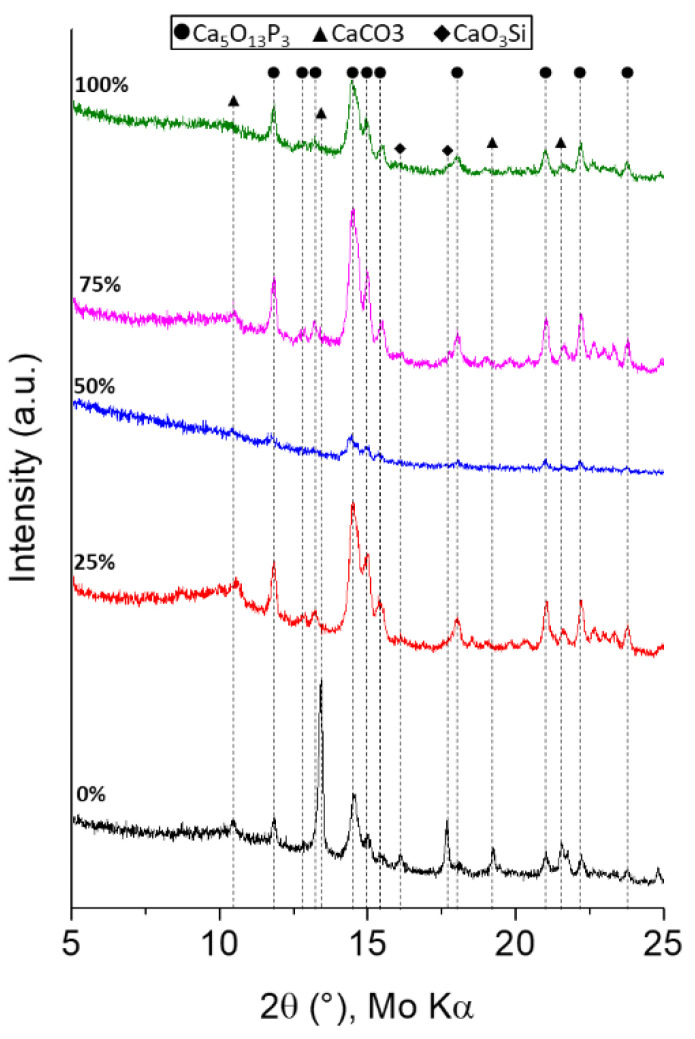
XRD patterns of the as-prepared materials via the sol–gel method. Black line 0%-group, red line 25%-group, blue line 50%-group, pink line 75%-group, green line 100%-group.

The x-ray diffraction (XRD) patterns of the as-prepared materials investigated within this work are shown in Figure 1. The patterns indicate that the samples are glass-ceramic materials. On the one hand, they are x-ray amorphous, since all of them show a broad hump centered at approximately 10° which is attributed to the amorphous silica network [26]. On the other hand, the XRD investigation revealed that crystallization had occurred, since diffraction peaks were attributed to calcium-content crystalline phases such as hydroxylapatite (Ca_5_(PO_4_)_3_OH, PDF 96-901-4314), calcite (CaCO_3_, PDF 96-901-6707) and wollastonite (CaSiO_3_, PDF 96-900-5779), which have also been identified in all of the patterns. The intensity and the shape of the crystalline peaks varies as a function of the theoretical (nominal) wt.% CaO used in the sol–gel approach. When the theoretical percentage is lower than ca. 40, the main crystalline phase detected is hydroxylapatite, while when the wt.% of CaO is higher than 40, besides the peaks related to hydroxylapatite, diffraction peaks attributed to calcium oxide and calcium silicate crystalline phases are also detected. Interestingly, the 50% group sample, which contained 32.1 wt% CaO, was found to be mainly X-ray amorphous, since its pattern only showed broad- and low-intensity hydroxylapatite diffraction peaks.

The XRD patterns of the calcinated samples do not show any diffraction peaks related to either calcium or sodium nitrate, indicating that both modifiers were successfully incorporated.

A summary of the results obtained from the particle-size distribution measurements is given in Table 3. Dv# is defined as the calculated equivalent spherical diameter, where # of the population lies below this value. Therefore, half of the distribution lies below Dv50, while 90 and 10 percent of the population lie below Dv90 and Dv10, respectively. Thus, we can make the following approximation: Dv50 is approximately the medium value, while the range between Dv10 and Dv90 is the particle dispersion (PD). Dv50 values lower than 500 nm were calculated for samples prepared with a certain wt.% of Na_2_O. Dv50 significantly increased when in the sample preparation all Na_2_O was replaced by CaO (Dv50 of 1400 nm). A similar trend was observed for particle dispersion. The sample prepared with 0 wt% of Na_2_O showed higher dispersion (PD of 5500 nm) than the samples prepared with Na_2_O, which displayed PD ranges from 280 nm to 619 nm.

For particles suspended in a fluid, the zeta potential is defined as the electrical potential difference between the liquid and the stationary layer of the fluid attached to the surface of the particle. It has been reported that negative zeta potential facilitates the attachment and proliferation of proteins and cells on the surface of the biomaterial when they are compared with surfaces with zeta potentials equal to or greater than zero mV [27,28,29,30]. When the as-synthesized BG particles were suspended in distilled water for 5 min (time needed to get stable measurement) they displayed negative zeta potential, as shown in Table 3. The zeta potential of the different materials exhibited an indirect relationship with the wt% of CaO used for preparing the glasses. Thus, 100 Na (lowest CaO wt%) displayed the highest zeta potential in absolute value (−42.3 mV ± 4.90 mV) while the zeta potential of 0 Na (highest CaO wt%) was found to show the lowest value (−22.9 mV ± 3.54 mV). The variations in the zeta potential with the pH are mainly attributed to the protonation/deprotonation of silanol sites on the surface of the material. Since it is a surface reaction, its magnitude (in absolute value) is related to the chemical composition of the glass, the particle size and the surrounding environment. Thus, we can conclude that it is mainly dependent on their chemical composition.

### 3.2. Ion Release from the As-Prepared BG Particles

**Figure 2:**
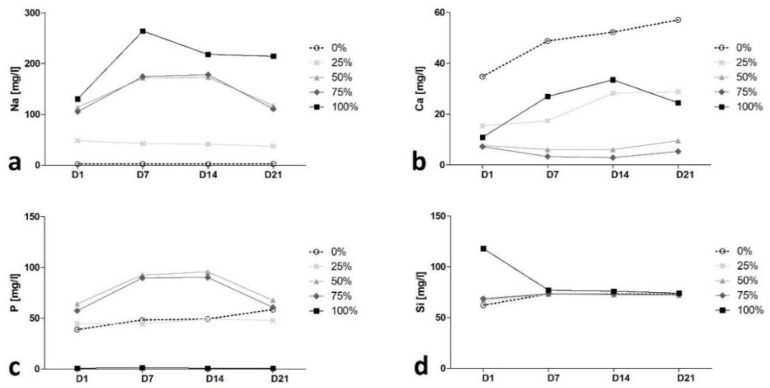
Ion release characteristics of the different BGs. Subgroup-specific release of Na ions (**a**), Ca ions (**b**), P ions (**c**) and Si ions (**d**) over time. Standard deviations are too small to be visible within the graph.

The dissolution study of the materials prepared within this work, upon exposure to distilled water over different soaking times, is of great interest to correlate the material ionic dissolution profiles with the results obtained during in vitro studies. Regarding the concentration of Na in the solutions (Figure 2a), all glasses showed the same trend: an increase in Na concentration over time, which was more pronounced during the first days of immersion. The relative Na concentration in the solution increased with Na substitution in the material over time. Thus, the highest concentration of Na was observed for the 100%-group while the 25%-group exhibited the lowest Na concentration in the solution during the whole experiment. Similar trends were observed concerning the release of Ca ions (Figure 2b), as the 0%-group showed the highest Ca concentration throughout the whole measurement period. Interestingly, the 100%-group (lowest nominal Ca content) showed an unexpectedly high Ca concentration in the solution as compared with the 75%-, 50%- and 25%-groups, which contained a higher nominal Ca content. This fact can be explained in terms of particle size. The 100%-group exhibited the lowest Dv50 value (Table 3) and therefore the highest surface area. Thus, it seems that Ca release depends not only on the nominal concentration of the ion in the material but also on the surface area.

The P ion concentration was found to be somewhat proportional to the P content of the different BGs (Figure 2c). The 0%- and 25%-groups showed mean P levels that were lower compared to the 50%- and 75%-groups. For the 100% group, the P ion concentration was zero over time. This fact may be because the release of P ions from the 100%-group granule occurred during the first 24 h of immersion. Thus, it could react with the surrounding Ca, forming calcium phosphate phases. Ion contents were best represented by the release characteristics and the mean levels of Si ions. As all BGs showed an almost identical Si content, apart from the early peak of the 100%-group, all BGs showed identical mean Si levels throughout the measurement period (Figure 2d).

Several ion concentrations decreased with increasing incubation time. No media changes were performed, and no medium was removed in order to observe the cumulated ion concentrations. Thus, it can be assumed that in some cases, after an initial ion release, precipitation occurred and led to a decrease in the accumulated ion concentration compared to earlier time points.

### 3.3. Na-Dependent pH-Increase

**Figure 3:**
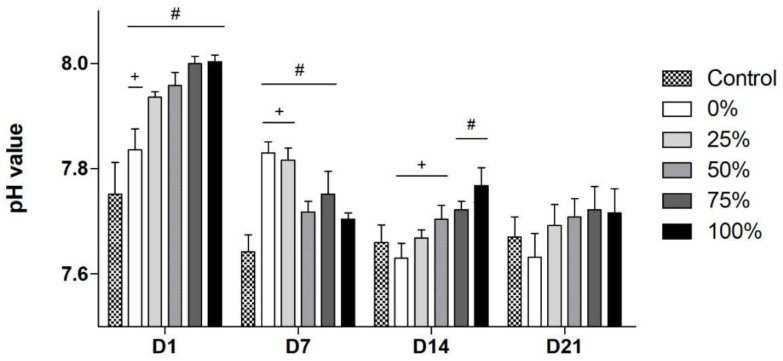
pH values for the different bioactive glass (BG) groups over time. (+) indicates significant differences to the original 45S5-BG composition (100% group); (#) indicates significant differences compared to the control group.

The baseline pH value of the cell- and BG-free CCM was found to be 7.48. All subgroups showed a similar trend: an increase in corresponding pH values in the early stages followed by a decrease and stabilization at the end of incubation (Figure 3). The highest absolute values were found for the 75%- and 100%-groups, reaching a pH of 8 on D1. Additionally, subgroups with higher Na contents also showed a faster increase to their maximum (D1) and a quicker, more noticeable decrease (D7) afterwards. The increase in pH values is caused by the ion exchange between the Na located on the surface of the glass and the hydrogen ions from the surrounding liquid [31]. Since it is a surface reaction, its evolution depends on the chemistry and specific surface area of the materials and on environmental conditions such as temperature, pressure and ionic strength. Here, since the variables related to the liquid media were constant during earlier exposure times, it seems that the differences found in the evolution of the pH are more closely related to the Na substitution in the BGs than to the size of their surface areas. As the reaction progresses, the process becomes more complicated, and other factors must be considered, such as the precipitation of new species that prevent the exit of sodium or the increase in the ionic strength of the liquid medium through the increasing concentration of ions from the mineralization of the materials. On D7, the pH normalization of the BG groups with high Na contents was ahead in time. Ion release and pH normalization were prolonged in the BG groups with lower Na contents by a more continuous ion release. The liquid media that experienced a lower pH variation (and therefore better resemble the physiological cell environment) throughout the whole incubation time were those treated with the 0%- and 25%- group. Although pH values at D21 were seen to be tending higher with increasing Na contents in the BGs, there were no statistically significant differences found between all subgroups and the control group.

### 3.4. Cell Viability in Relation to the Different Na Contents

**Figure 4:**
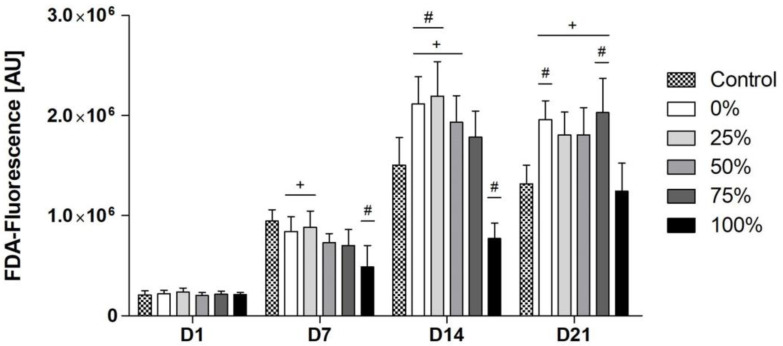
FDA fluorescence for the different BG groups over time. (+) indicates significant differences to the original 45S5-BG composition (100% group); (#) indicates significant differences compared to the control group.

Whilst the control group was most viable on D7, viability in the 0%-, 25%-, 50% and 75%-groups exceeded the viability of the control group, with the 0%- and the 25%-group showing significantly higher viability compared to the control group from D14 on (Figure 4). However, the viability of cells in the 100%-group remained significantly impaired compared to the control group and the 0%-, 25%- and 50%-groups. On D21, the 0%- and 75%-group showed significantly higher viability compared to the control group, while viability in all BG groups was significantly higher compared to the 100%-group. Cell viability tests were carried out as direct co-cultures with a concentration of 1 mg/mL for the different BGs.

### 3.5. ALP Activity

**Figure 5:**
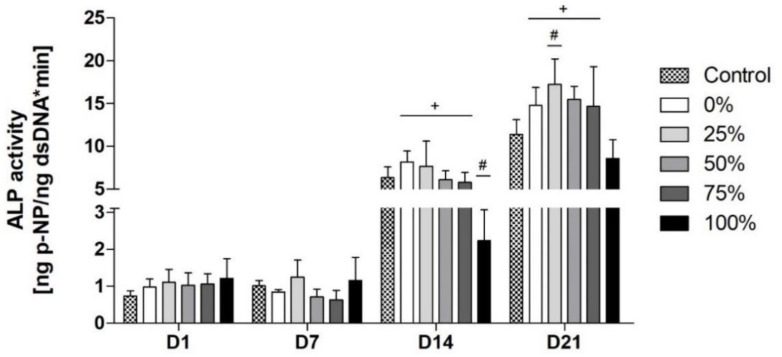
Alkaline Phosphatase (ALP) activity for the different bioactive glass (BG) groups over time. (+) indicates significant differences to the original 45S5-BG composition (100% group); (#) indicates significant differences compared to the control group.

As expected, a considerable increase in ALP activity was noticeable for all groups from D7 to D14 since this period is considered to be critical in terms of cellular differentiation towards osteoblasts and thus for an increase in ALP activity (Figure 5). Interestingly, at D14 significantly lower values were found for the 100%-group compared to all other groups, including the control group. The other groups with reduced Na contents did not differ significantly from the control group but showed slightly increased ALP activity. On D21, the 25%-group was the only group that showed significantly higher ALP activity levels than the control group and the highest absolute values during the whole incubation period.

### 3.6. qPCR of Genes Encoding for ECM Proteins

**Figure 6:**
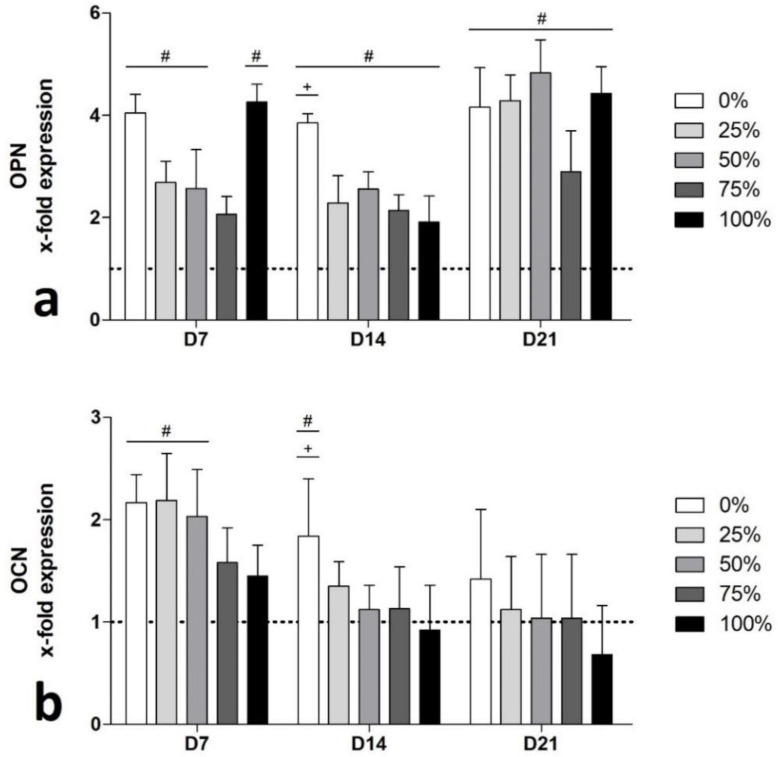
Osteogenic differentiation during the incubation period (D7-D21) represented by gene expression of osteopontin (*OPN*, **a**) and osteocalcin (*OCN*, **b**). Values are normalized to the control group (indicated by the dotted line) and shown as means with standard deviation. (+) indicates significant differences from the original 45S5-BG composition (100% group); (#) indicates significant differences compared to the control group.

Regarding *OPN* expression, no significant differences were detectable between the BG groups on D7 (Figure 6a). However, despite the 75%-group, all groups showed significantly higher *OPN* gene expression compared to the control group. At D14 and D21, all BG groups significantly outperformed the control, indicating a strong pro-osteogenic stimulation provided by the BGs. On D14, the Na-free BG group even significantly outperformed the original 45S5-BG composition.

The expression of the *OCN* gene was significantly stimulated by the 0%-, 25%- and 50%-groups at D7 compared to the control group (Figure 6b), whilst no significant differences were detectable between the BG groups. On D14, only the 0%-group showed significantly higher *OCN* expression levels compared to the 100%-group and to the control group, whilst on D21, the expression levels of *OCN* in the BG groups approximated the level of the control.

## 4. Discussion

Due to their high initial bioreactivity, which is affiliated with a burst release of Na ions in in vitro cell culture settings, BGs might develop challenging surroundings for cells [16]. Therefore, significant efforts have been undertaken to improve the biological properties of BGs [1,6,7,11,12,32,33,34]. Since these possibly harmful effects have been attributed (at least in part) to the initial Na burst release, e.g., by causing a pH elevation, a reduction in Na content of BGs based on the 45S5-BG composition might improve cytocompatibility [11,13,14,15]. Therefore, the aim of this study was on the one hand to identify the differences in physicochemical properties between glass-ceramic materials with different Na content prepared by the sol–gel method. Differences in physicochemical features such as crystalline phases, particle size distribution and surface charge are known to influence material dissolution, cell/protein attachment and subsequent mechanisms leading to hydroxycarbonate layer formation. On the other hand, differences in cell viability and cellular osteogenic differentiation as well as the expression of genes encoding for ECM proteins were assessed using BMSCs that were exposed to the respective sol–gel-derived Na-reduced BGs based on the original 45S5-BG composition.

In the present work, it has been shown that the 45S5-BG-based materials with different Na substitution prepared by the sol–gel method and calcinated at 700 °C exhibit partial crystallization. Thus, the as-prepared materials are glass-ceramics rather than pure glasses. However, the substitution of Na_2_O by CaO influences the amorphous resistance of the materials (glass-ceramics) and their mineralogical structure. Regarding the amorphous resistance of the materials, we observed that when the nominal wt% CaO is ca. 40 the material is mainly amorphous. The further we move away from that percentage, the lower is the resistance to crystallization of the material. The crystalline phases are mainly governed by the most abundant modifier (Ca or Na) in the sample. The Na-rich materials exhibit mainly hydroxylapatite crystals with traces of CaSiO_3_, while in the Ca-rich powders the crystallization of Ca-oxides and silicates besides Ca-phosphates (hydroxylapatite) was mainly found. The incorporation of different crystalline phases with different reactivity in biological environments on the glasses has been reported as an alternative approach to controlling dissolution rates and therefore to controlling increases in physiological pH [18].

Powders derived from the sol–gel method are theoretically characterized by having primary particle sizes (size of unbounded particles) in the nanometer range. However, unless their surface is properly treated, these nanoparticles are agglomerated. The degree of agglomeration depends on different factors and can affect its reactivity. In our study, upon exposure to distilled water for no longer than 5 min, the particles exhibited agglomeration, which can be correlated with the amount of calcium. Interestingly, we observed that both the average particle size and the particle agglomeration degree significantly increased when the CaO wt% used for preparing the material exceeded approximately 40 wt%. Thus, when the nominal percentage of CaO is greater than 40, one can expect that the specific surface areas of the particles will be dramatically decreased, providing a less exposed surface for dissolution and, consequently, decreasing the rate of processes such as the deposition of a Ca-P layer, degradation and resorption. The surface of the as-prepared powders is negative independent of their composition due to the silanol groups developed upon exposure to water [35]. The density of negative charges depends directly on the sodium content in such a way that the higher the nominal wt% of Na_2_O used, the higher the density of negative charges on the surface of the particle. It should be noted that when a material is implanted, the proteins from the blood or lymph have to adhere to the material. This step is decisive for the material to actually develop bioactivity. Otherwise, the material is inert, and tissue regeneration will not take place. A necessary but not sufficient condition for such attachment to occur is that the surface of the materials must be negatively charged.

In particular, we did not perform specific assessments to demonstrate actual bioreactivity or to explore further physical properties (such as textural properties) of the as-prepared materials, since the focus of this present work was on the impact of those materials on the cellular environment. Furthermore, no chemical analysis with regards to the real compositions and resulting Na contents of the as-synthesized BGs compared to the nominal compositions were carried out.

Regarding the ion release characteristics, a ranking of the different BGs was evident (Table 1, Figure 2). The ion concentration mainly corresponded to and represented the content of the specific ions. The only exception was found for Ca. Its release not only depends on its theoretical initial concentration in the material but also on its particle size and agglomeration state. Most relevantly, we have found that a rise in physiological pH is more closely related to the Na substitution in the BGs than to other factors such as specific surface area (approximated by the values of the particle average size and agglomeration) or the type of crystalline phases in the material. The negative effect of Na ions and positive effects of Ca ions on cell viability and proliferation have been shown in the past [13,15,36]. Based on the release characteristics of the different BGs concerning all the different ions, our results suggest that BGs with a Na content reduced to 25% to 50% of the original 45S5-BG might be beneficial and lead to a superior outcome due to the lower levels of Na and the medium to high amount of Ca ions (Table 1, Figure 2).

In in vitro cell culture settings, the potential cytotoxicity of high Na contents in BGs has been explained partially by a consecutive and dramatic pH elevation [1,13,14,15]. Kansal et al. have measured the pH values during dissolution of different BGs with varying Na contents in the MgO–Na_2_O–CaO–P_2_O_5_–SiO_2_–CaF_2_ system, observing lower and more physiological pH values of BGs with a reduced Na content [15]. These lower pH values were supporting biocompatibility and cell proliferation [15]. Our results compare to these findings (Figure 3). In the present study, all BGs showed an increase in the pH in the early stages followed by a decrease and stabilization at the end. In general, a high Na content led to an accelerated increase and to higher absolute pH values compared to BGs with a low Na content. More physiological pH values were seen for BGs with a low Na content. Moreover, the observed high values and the accelerated increase could both exert cytotoxic effects and thus reduce cytocompatibility equally. As Kansal et al. have measured pH values during the first 125 h, a comparison of our results with respect to the decrease in and stabilization of pH values during the late stages is not possible [15]. Furthermore, the most pronounced increase in pH was observed at D1, whilst pH values showed a tendency towards normalization to the physiological pH from then on until D21. This finding corresponds well to the idea that the initial burst release of ions in combination with its associated increase in local pH values is responsible for the potential cytotoxicity of BGs when introduced to cell culture settings [16]. This also explains why preconditioning approaches in settings using 45S5-BG particles in cell cultures are effective when performed for 24 h but do not show further benefits after longer preincubation periods: as shown in a study conducted by our group, the viability of human BMSCs improved when cultivated in the presence of 45S5-BG particles that were passivated for 24 h compared to shorter passivation periods [37]. A passivation of 72 h compared to a 24 h period led neither to a further normalization of the pH value nor to a significant improvement in BMSC viability [37].

It has been shown that the content of Na has an influence on cell viability and proliferation by various authors [11,13,14,15]. Our data suggest that Na-reduced BGs support cell viability (Figure 4). Brito et al. report on similar effects analyzing a series of Na-free BGs also using human BMSCs [11]. The authors report that Na-deficient BGs show superior properties with respect to cell viability and proliferation using a WST-1 proliferation assay [11]. However, the analyzed BGs that have been compared to the 45S5-BG composition were not Na-free variants of 45S5-BG but BGs from other systems. Thus, a direct comparability between the BGs and 45S5-BG was not possible due to the differences regarding the presence of other ions such as magnesium, which was part of all analyzed BGs except for 45S5-BG [11]. In addition, Kansal et al. have prepared different BGs in the MgO−Na_2_O–CaO–P_2_O_5_–SiO_2_–CaF_2_ system with varying Na contents using the melt-quenching technique and tested these with respect to cell proliferation [15]. The authors showed that the incorporation of Na decreases cell viability to a minimum after 14 days of incubation, even for the BG with the lowest Na content [15]. Vice versa, Zhao et al. synthesized and tested pH-neutral BGs in comparison to 45S5-BG and beta-tricalcium phosphate in vitro and in vivo [38]. In both settings, the pH-neutral BGs proved to be superior with respect to cell proliferation and osteogenic and angiogenic properties [38]. To our knowledge, so far, no other study consistently assessed the effect of different BGs based on the composition of 45S5-BG with progressively reduced Na contents with respect to cell viability using osteogenic cells.

ALP is an early marker of cellular osteogenic differentiation [39]. As osteoblast precursor cells shift during differentiation from ALP production to the production of ECM proteins, ALP activity levels are expected to increase from D7 until D14 and consecutively decrease during further differentiation [39,40,41]. However, during our study there was a delayed onset but a consistent course over time and between the different BG groups. The 25%-group in particular showed enhanced osteogenic differentiation, with significantly higher values than the control group (Figure 5). Similar effects were reported previously [11,32]. Goel et al. and Brito et al. tested custom-made alkali-free BGs using the melt-quenching technique and compared these compositions to 45S5-BG [11,32]. The authors also assessed osteogenic differentiation, measuring ALP activity during incubation [11,32]. They concluded that the tested alkali-free compositions significantly increased osteogenic differentiation compared to the culture plastic control and could therefore be seen as equivalent or even superior alternatives to 45S5-BG [11,32]. However, relevant methodical differences are noticeable [11,32]. Goel et al. used rodent BMSC [32]. It is known that stem cells obtained from different species or locations show different properties in vitro [7,42,43,44,45]. Furthermore, for measurements of ALP activity, the authors used osteogenic differentiation media for cell cultivation [32]. By contrast, osteogenic differentiation in our study could be induced by the presence of the different BGs only, as no other potential stimuli were used. Brito et al. analyzed the ALP activity and osteogenic differentiation qualitatively, whereas a quantitative assessment was realized in our study [11]. In their studies, Goel et al. and Brito et al. did not compare Na-reduced variants of 45S5-BG to the original 45S5-BG composition but to BGs from other systems that had a comparably lower Na content—being one of the major differences between the presented study and the cited ones [11,32]. Moreover, within a certain range of concentration, Na seems to have a stimulating effect on ALP activity [46]. Not only Na but other therapeutically active ions released from different BGs show concentration-dependent effects on osteogenic differentiation on a cellular level represented by ALP activity [4,7,10,47]. Therefore, BGs with moderate Na contents assessed in this study seem to affect cellular osteogenic differentiation positively.

After the phase of early osteogenic differentiation, gene expression levels of *OPN* and *OCN* increase as the ECM production provided by osteoblasts is anticipated to increase between D14 and D28 of cell culture [39,48,49]. As *OCN* is known as a bone specific protein and marker of late stage differentiation, gene expression levels are expected to increase after D14 [39,49,50]. Expression levels of *OPN* were significantly elevated on D7 for all groups, with exception of the 75%-group, and substantially constant until D21 (Figure 6). Corresponding to the accelerated/early increase in *OPN* expression levels, an early increase in *OCN* expression was found as well. Furthermore, as expressions levels decreased over time with increasing Na content, Na seemed to inhibit the genes encoding for relevant ECM members after an initial stimulation (Figure 6).

The general positive effects of Na reduction on cell viability, cell proliferation and osteogenic differentiation might be further enhanced by doping the Na-reduced BGs with other therapeutically active ions [4,6]. There is a vast number of candidate ions that have been demonstrated to enhance the osteogenic properties of BGs, their biocompatibility or generally alter their biological properties. Some well-known examples are boron, which is known to be pro-angiogenic, or zinc, which favors calcification [51,52]. Further, less well-known ions are being investigated currently, such as Molybdenum, which has been shown to enhance the osteogenic differentiation of bone precursor cells, or Cerium, which has been proven to be antioxidative [53,54,55].

The main limitations of this work are the lack of chemical analysis of the real BG compositions and in particular the measurement and assessment of the effective content of Na in the synthesized BGs. However, considering the fact that nominal compositions are approximations to the real values, we assume that the real values for Na contents might be lower than expected but that there are still proportional ratios between the nominal and real compositions for all as-synthesized Na-reduced BG compositions studied in this work; this would allow for relevant conclusions on the effect of Na contents in BGs and the resulting effect on osteogenic properties and cytocompatibility.

## 5. Conclusions

In this study, Na-reduced variants of the well-known 45S5-BG composition were successfully produced via the sol–gel route and have been compared regarding cytocompatibility and osteogenic differentiation in a static cell culture approach. It was observed that the reduction in the Na portion enhances the BGs’ cytocompatibility. However, the presence of Na ions has a direct concentration-dependent impact on cellular osteogenic differentiation, as shown by alterations in ALP activity and a less pronounced impact on the expression of genes encoding for relevant protein members of the ECM. Based on the findings of this study, due to their favorable cytocompatibility and osteogenic properties the 25% and 50% Na-reduced variants of the original 45S5-BG composition should be considered for further experimental investigation. Future studies should analyze BG–cell interaction in greater detail and might assess the in vivo performance of these most promising Na-reduced 45S5-BG variants. Furthermore, the incorporation of further ions that have been shown to enhance cellular osteogenic differentiation or ECM development can be considered in order to further improve the osteogenic properties of Na-reduced 45S5-BG variants.

## Figures and Tables

**Table 1:** Nominal composition of powders based on the 45S5 composition (100%-group) and with Na substitution for Ca (75%-, 50%-, 25%- and 0%-group).

[wt% Na]	SiO_2_	Na_2_O	CaO	P_2_O_5_
100% (original 45S5-BG)	45.0	24.5	24.5	6.0
75%	45.0	18.3	27.5	9.2
50%	45.0	12.2	32.1	10.7
25%	45.0	6.1	36.7	12.2
0%	45.0	0.0	41.2	13.8

**Table 2:** Primers used for qPCR. 14-3-3 protein zeta/delta (*YWHAZ*; reference gene), osteocalcin (*OCN*), osteopontin (*OPN*).

Gene	Forward (5′–3′)	Reverse (3′–5′)
YWHAZ	TGC TTG CAT CCC ACA GAC TA	AGG CAG ACA ATG ACA GAC CA
OCN	ACC GAG ACA CCA TGA GAC CC	GCT TGG ACA CAA AGG CTG CAC
OPN	GCT AAA CCC TGA CCC ATC TC	ATA ACT GTC CTT CCC ACG GC

**Table 3:** Average particle size (Dv50), particle size distribution (Dv10, Dv90 and PD) and Z- potential (ZP) of the as-synthesized different BGs (specified by their Na-content in wt%), suspended in distilled water at 25 °C. The measurements were performed in times no longer than 5 min.

[wt% Na]	Dv50 (nm)	Dv10 (nm)	Dv90 (nm)	PD (nm)	ZP (mV)
100%	226	67	347	280	−42.3 ± 4.90
75%	496	137	756	619	−30.5 ± 5.96
50%	414	178	655	477	−38.1 ± 5.25
25%	356	59	489	430	−26.6 ± 4.30
0%	1400	776	5500	4724	−22.9 ± 3.54

## Data Availability

The data presented in this study are available on request from the corresponding author.

## References

[1] Baino F., Hamzehlou S., Kargozar S. (2018). Bioactive Glasses: Where Are We and Where Are We Going?. J. Funct. Biomater..

[2] Wang W., Yeung K.W.K. (2017). Bone grafts and biomaterials substitutes for bone defect repair: A review. Bioact. Mater..

[3] Dimitriou R., Mataliotakis G.I., Angoules A.G., Kanakaris N.K., Giannoudis P.V. (2011). Complications following autologous bone graft harvesting from the iliac crest and using the RIA: A systematic review. Injury.

[4] Hoppe A., Guldal N.S., Boccaccini A.R. (2011). A review of the biological response to ionic dissolution products from bioactive glasses and glass-ceramics. Biomaterials.

[5] Jones J.R. (2013). Review of bioactive glass: From Hench to hybrids. Acta Biomater..

[6] Rahaman M.N., Day D.E., Bal B.S., Fu Q., Jung S.B., Bonewald L.F., Tomsia A.P. (2011). Bioactive glass in tissue engineering. Acta Biomater..

[7] Westhauser F., Hohenbild F., Arango-Ospina M., Schmitz S.I., Wilkesmann S., Hupa L., Moghaddam A., Boccaccini A.R. (2020). Bioactive Glass (BG) ICIE16 Shows Promising Osteogenic Properties Compared to Crystallized 45S5-BG. Int. J. Mol. Sci..

[8] Hench L.L., Paschall H.A. (1973). Direct chemical bond of bioactive glass-ceramic materials to bone and muscle. J. Biomed. Mater. Res..

[9] Westhauser F., Karadjian M., Essers C., Senger A.S., Hagmann S., Schmidmaier G., Moghaddam A. (2019). Osteogenic differentiation of mesenchymal stem cells is enhanced in a 45S5-supplemented beta-TCP composite scaffold: An in-vitro comparison of Vitoss and Vitoss BA. PLoS ONE.

[10] Xynos I.D., Edgar A.J., Buttery L.D., Hench L.L., Polak J.M. (2000). Ionic products of bioactive glass dissolution increase proliferation of human osteoblasts and induce insulin-like growth factor II mRNA expression and protein synthesis. Biochem. Biophys. Res. Commun..

[11] Brito A.F., Antunes B., Dos Santos F., Fernandes H.R., Ferreira J.M.F. (2017). Osteogenic capacity of alkali-free bioactive glasses. In vitro studies. J. Biomed. Mater. Res. Part B Appl. Biomater..

[12] Karadjian M., Essers C., Tsitlakidis S., Reible B., Moghaddam A., Boccaccini A.R., Westhauser F. (2019). Biological Properties of Calcium Phosphate Bioactive Glass Composite Bone Substitutes: Current Experimental Evidence. Int. J. Mol. Sci..

[13] Wallace K.E., Hill R.G., Pembroke J.T., Brown C.J., Hatton P.V. (1999). Influence of sodium oxide content on bioactive glass properties. J. Mater. Sci. Mater. Med..

[14] Brauer D.S., Karpukhina N., O’Donnell M.D., Law R.V., Hill R.G. (2010). Fluoride-containing bioactive glasses: Effect of glass design and structure on degradation, pH and apatite formation in simulated body fluid. Acta Biomater..

[15] Kansal I., Reddy A., Muñoz F., Choi S.-J., Kim H.-W., Tulyaganov D.U., Ferreira J.M.F. (2014). Structure, biodegradation behavior and cytotoxicity of alkali-containing alkaline-earth phosphosilicate glasses. Mater. Sci. Eng. C.

[16] Ciraldo F.E., Boccardi E., Melli V., Westhauser F., Boccaccini A.R. (2018). Tackling bioactive glass excessive in vitro bioreactivity: Preconditioning approaches for cell culture tests. Acta Biomater..

[17] Baino F., Fiume E., Miola M., Verné E. (2018). Bioactive sol-gel glasses: Processing, properties, and applications. Int. J. Appl. Ceram. Technol..

[18] Sepulveda P., Jones J.R., Hench L.L. (2001). Characterization of melt-derived 45S5 and sol-gel-derived 58S bioactive glasses. J. Biomed. Mater. Res..

[19] Sepulveda P., Jones J.R., Hench L.L. (2002). In vitro dissolution of melt-derived 45S5 and sol-gel derived 58S bioactive glasses. J. Biomed. Mater. Res..

[20] Arcos D., Vallet-Regí M. (2010). Sol-gel silica-based biomaterials and bone tissue regeneration. Acta Biomater..

[21] Pirayesh H., Nychka J.A. (2013). Sol–Gel Synthesis of Bioactive Glass-Ceramic 45S5 and its in vitro Dissolution and Mineralization Behavior. J. Am. Ceram. Soc..

[22] Delgado A.V., González-Caballero F., Hunter R.J., Koopal L.K., Lyklema J. (2005). Measurement and Interpretation of Electrokinetic Phenomena (IUPAC Technical Report). Pure Appl. Chem..

[23] Reible B., Schmidmaier G., Moghaddam A., Westhauser F. (2018). Insulin-Like Growth Factor-1 as a Possible Alternative to Bone Morphogenetic Protein-7 to Induce Osteogenic Differentiation of Human Mesenchymal Stem Cells in Vitro. Int. J. Mol. Sci..

[24] Reible B., Schmidmaier G., Prokscha M., Moghaddam A., Westhauser F. (2017). Continuous stimulation with differentiation factors is necessary to enhance osteogenic differentiation of human mesenchymal stem cells in-vitro. Growth Factors.

[25] Widholz B., Tsitlakidis S., Reible B., Moghaddam A., Westhauser F. (2019). Pooling of Patient-Derived Mesenchymal Stromal Cells Reduces Inter-Individual Confounder-Associated Variation without Negative Impact on Cell Viability, Proliferation and Osteogenic Differentiation. Cells.

[26] Khouchaf L., Boulahya K., Das P.P., Nicolopoulos S., Kis V.K., Lábár J.L. (2020). Study of the Microstructure of Amorphous Silica Nanostructures Using High-Resolution Electron Microscopy, Electron Energy Loss Spectroscopy, X-ray Powder Diffraction, and Electron Pair Distribution Function. Materials.

[27] Cooper J.J., Hunt J.A. (2006). The Significance of Zeta Potential in Osteogenesis. Society for Biomaterials. Transactions of the 31st Annual Meeting for Biomaterials Pittsburgh.

[28] Krajewski A., Malavolti R., Piancastelli A. (1996). Albumin adhesion on some biological and non-biological glasses and connection with their Z-potentials. Biomaterials.

[29] Smeets R., Kolk A., Gerressen M., Driemel O., Maciejewski O., Hermanns-Sachweh B., Riediger D., Stein J.M. (2009). A new biphasic osteoinductive calcium composite material with a negative Zeta potential for bone augmentation. Head Face Med..

[30] Teng N.C., Nakamura S., Takagi Y., Yamashita Y., Ohgaki M., Yamashita K. (2001). A new approach to enhancement of bone formation by electrically polarized hydroxyapatite. J. Dent. Res..

[31] Blochberger M., Hupa L., Brauer D. (2015). Influence of zinc and magnesium substitution on ion release from Bioglass 45S5 at physiological and acidic pH. Biomed. Glas..

[32] Goel A., Kapoor S., Rajagopal R.R., Pascual M.J., Kim H.W., Ferreira J.M. (2012). Alkali-free bioactive glasses for bone tissue engineering: A preliminary investigation. Acta Biomater..

[33] Schmitz S.I., Widholz B., Essers C., Becker M., Tulyaganov D.U., Moghaddam A., Gonzalo de Juan I., Westhauser F. (2020). Superior biocompatibility and comparable osteoinductive properties: Sodium-reduced fluoride-containing bioactive glass belonging to the CaO-MgO-SiO_2_ system as a promising alternative to 45S5 bioactive glass. Bioact. Mater..

[34] Sergi R., Bellucci D., Salvatori R., Maisetta G., Batoni G., Cannillo V. (2019). Zinc containing bioactive glasses with ultra-high crystallization temperature, good biological performance and antibacterial effects. Mater. Sci. Eng. C.

[35] Behrens S.H., Grier D.G. (2001). The charge of glass and silica surfaces. J. Chem. Phys..

[36] Maeno S., Niki Y., Matsumoto H., Morioka H., Yatabe T., Funayama A., Toyama Y., Taguchi T., Tanaka J. (2005). The effect of calcium ion concentration on osteoblast viability, proliferation and differentiation in monolayer and 3D culture. Biomaterials.

[37] Hohenbild F., Arango-Ospina M., Moghaddam A., Boccaccini A.R., Westhauser F. (2020). Preconditioning of Bioactive Glasses before Introduction to Static Cell Culture: What Is Really Necessary?. Methods Protoc..

[38] Zhao H., Liang G., Liang W., Li Q., Huang B., Li A., Qiu D., Jin D. (2020). In vitro and in vivo evaluation of the pH-neutral bioactive glass as high performance bone grafts. Mater. Sci. Eng. C.

[39] Birmingham E., Niebur G.L., McHugh P.E., Shaw G., Barry F.P., McNamara L.M. (2012). Osteogenic differentiation of mesenchymal stem cells is regulated by osteocyte and osteoblast cells in a simplified bone niche. Eur. Cells Mater..

[40] Aubin J.E. (2001). Regulation of osteoblast formation and function. Rev. Endocr. Metab. Disord..

[41] Huang Z., Nelson E.R., Smith R.L., Goodman S.B. (2007). The Sequential Expression Profiles of Growth Factors from Osteroprogenitors to Osteoblasts In Vitro. Tissue Eng..

[42] Weinberger L., Ayyash M., Novershtern N., Hanna J.H. (2016). Dynamic stem cell states: Naive to primed pluripotency in rodents and humans. Nat. Rev. Mol. Cell Biol..

[43] Baboolal T.G., Boxall S.A., El-Sherbiny Y.M., Moseley T.A., Cuthbert R.J., Giannoudis P.V., McGonagle D., Jones E. (2014). Multipotential stromal cell abundance in cellular bone allograft: Comparison with fresh age-matched iliac crest bone and bone marrow aspirate. Regen. Med..

[44] Fragkakis E.M., El-Jawhari J.J., Dunsmuir R.A., Millner P.A., Rao A.S., Henshaw K.T., Pountos I., Jones E., Giannoudis P.V. (2018). Vertebral body versus iliac crest bone marrow as a source of multipotential stromal cells: Comparison of processing techniques, tri-lineage differentiation and application on a scaffold for spine fusion. PLoS ONE.

[45] Herrmann M., Hildebrand M., Menzel U., Fahy N., Alini M., Lang S., Benneker L., Verrier S., Stoddart M.J., Bara J.J. (2019). Phenotypic Characterization of Bone Marrow Mononuclear Cells and Derived Stromal Cell Populations from Human Iliac Crest, Vertebral Body and Femoral Head. Int. J. Mol. Sci..

[46] Utida S. (1967). Effect of Sodium Chloride on Alkaline Phosphatase Activity in Intestinal Mucosa of the Rainbow Trout. Proc. Jpn. Acad..

[47] Xynos I.D., Hukkanen M.V.J., Batten J.J., Buttery L.D., Hench L.L., Polak J.M. (2000). Bioglass ^®^45S5 Stimulates Osteoblast Turnover and Enhances Bone Formation In Vitro: Implications and Applications for Bone Tissue Engineering. Calcif. Tissue Int..

[48] Sodek J., Chen J., Nagata T., Kasugai S., Todescan R., Li I.W.S., Kim R.H. (1995). Regulation of Osteopontin Expression in Osteoblasts. Ann. N. Y. Acad. Sci..

[49] Wang L., Li Z.Y., Wang Y.P., Wu Z.H., Yu B. (2015). Dynamic Expression Profiles of Marker Genes in Osteogenic Differentiation of Human Bone Marrow-derived Mesenchymal Stem Cells. Chin. Med. Sci. J..

[50] Miron R.J., Zhang Y.F. (2012). Osteoinduction: A Review of Old Concepts with New Standards. J. Dent. Res..

[51] Westhauser F., Widholz B., Nawaz Q., Tsitlakidis S., Hagmann S., Moghaddam A., Boccaccini A.R. (2019). Favorable angiogenic properties of the borosilicate bioactive glass 0106-B1 result in enhanced in vivo osteoid formation compared to 45S5 Bioglass. Biomater. Sci..

[52] Westhauser F., Decker S., Nawaz Q., Rehder F., Wilkesmann S., Moghaddam A., Kunisch E., Boccaccini A.R. (2021). Impact of Zinc- or Copper-Doped Mesoporous Bioactive Glass Nanoparticles on the Osteogenic Differentiation and Matrix Formation of Mesenchymal Stromal Cells. Materials.

[53] Decker S., Kunisch E., Moghaddam A., Renkawitz T., Westhauser F. (2021). Molybdenum trioxide enhances viability, osteogenic differentiation and extracellular matrix formation of human bone marrow-derived mesenchymal stromal cells. J. Trace Elem. Med. Biol..

[54] Westhauser F., Rehder F., Decker S., Kunisch E., Moghaddam A., Zheng K., Boccaccini A.R. (2021). Ionic dissolution products of Cerium-doped bioactive glass nanoparticles promote cellular osteogenic differentiation and extracellular matrix formation of human bone marrow derived mesenchymal stromal cells. Biomed. Mater..

[55] Zheng K., Torre E., Bari A., Taccardi N., Cassinelli C., Morra M., Fiorilli S., Vitale-Brovarone C., Iviglia G., Boccaccini A.R. (2020). Antioxidant mesoporous Ce-doped bioactive glass nanoparticles with anti-inflammatory and pro-osteogenic activities. Mater. Today Bio.

